# Feline leishmaniosis: hematological and biochemical analysis

**DOI:** 10.1590/S1984-29612023035

**Published:** 2023-06-26

**Authors:** Diogo Tiago da Silva, Maria Luana Alves, Júlio Cesar Pereira Spada, João Augusto Franco Leonel, Geovanna Vioti, Julia Cristina Benassi, Valéria Maria Lara Carregaro, Maria Fernanda Alves-Martin, Wilma Aparecida Starke-Buzetti, Trícia Maria Ferreira de Sousa Oliveira

**Affiliations:** 1 Laboratório de Medicina Veterinária Preventiva Aplicada, Departamento de Medicina Veterinária, Faculdade de Zootecnia e Engenharia de Alimentos, Universidade de São Paulo - USP, Pirassununga, SP, Brasil; 2 Programa de Pós-Graduação em Epidemiologia Experimental Aplicada às Zoonoses, Departamento de Medicina Veterinária Preventiva e Saúde Pública, Faculdade de Medicina Veterinária e Zootecnia, Universidade de São Paulo - USP, Pirassununga, SP, Brasil; 3 Departamento de Biologia e Zootecnia, Escola de Engenharia, Universidade Estadual Paulista - UNESP, Ilha Solteira, SP, Brasil

**Keywords:** Cats, hyperproteinemia, hypoalbuminemia, Leishmania infantum, thrombocytopenia, Gatos, hiperproteinemia, hipoalbuminemia, Leishmania infantum, trombocitopenia

## Abstract

One hundred and sixty-six cats from two animal shelters were subjected to enzyme-linked immunosorbent assay (ELISA), indirect immunofluorescence antibody test (IFAT), conventional polymerase chain reaction (cPCR), quantitative PCR (qPCR) and parasitological tests (PA) for the diagnosis of *Leishmania* spp. Among them, 15% (25/166), 53.6% (89/166), 3.6% (06/166) and 1.8% (03/166) were positive by ELISA, IFAT, both PCRs and PA, respectively. The sequencing of ITS-1 PCR amplicons revealed a 100% match with *Leishmania infantum*. After the *Leishmania* spp. survey, 12 cats were selected and divided into two groups for clinical, hematological, and biochemical analysis: six *L. infantum* positive cats (G1) and six *Leishmania* spp. negative cats (G2). All the cats were negative for feline immunodeficiency virus (FIV) and feline leukemia virus (FeLV). A statistical analysis indicated significantly low platelet counts and significant hyperproteinemia associated with hypoalbuminemia in positive cats (p<0.05). Our results suggest that in endemic areas, cats with clinical signs of feline leishmaniosis (such as skin lesions, weight loss and/or enlarged lymph nodes) and that exhibit hematological and biochemical changes, such as low platelet counts and hyperproteinemia with hypoalbuminemia, should be tested for *Leishmania* spp. infection.

## Resumo

Cento e sessenta e seis gatos de dois abrigos foram submetidos ao diagnóstico de *Leishmania* spp. por ensaio imunoenzimático (ELISA), imunofluorescência indireta (RIFI), reação em cadeia pela polimerase convencional (cPCR) e quantitativa (qPCR) e métodos parasitológicos (PA). Destes, 15% (25/166), 53,6% (89/166), 3,6% (06/166) e 1,8% (03/166) foram positivos por ELISA, RIFI, as duas PCRs e PA, respectivamente. O sequenciamento dos produtos amplificados da PCR ITS-1 foi 100% idêntico à *Leishmania infantum*. Após o inquérito, 12 gatos foram selecionados para compor dois grupos para análises de hematologia e bioquímica: 6 gatos positivos para *L. infantum* (G1) e 6 gatos *Leishmania* spp. negativos (G2). Todos os gatos foram negativos para o vírus da imunodeficiência felina (FIV) e o da leucemia felina (FeLV). Foi observada uma diminuição na contagem de plaquetas e uma hiperproteinemia e hipoalbuminemia significativas em gatos positivos (p<0,05). Esses resultados sugerem que, em áreas endêmicas, os gatos com sinais clínicos de leishmaniose felina (tais como lesões dermatológicas, perda de peso e/ou linfonodos aumentados), associados a alterações hematológicas e bioquímicas, como contagem reduzida de plaquetas e hiperproteinemia com hipoalbuminemia, devem ser testados para leishmaniose felina.

## Introduction

Leishmaniosis comprises a group of complex diseases involving a wide range of parasites, vectors of phlebotomine sand flies and vertebrate hosts in endemic areas (DebRoy et al., 2017; Rossi & Fasel, 2018; Solano-Gallego et al., 2017). In Brazil cutaneous (CL) and visceral leishmaniosis (VL) are endemic diseases. In this country, CL is caused by various *Leishmania* spp., while VL is associated mainly with infection by *Leishmania infantum* (Brasil, 2021)

Dogs are considered the main domestic reservoir of VL in the zoonotic cycle of the disease and, together with the VL vectors (mainly *Lutzomyia longipalpis* but also *Lutzomyia* c*ruzi* and *Lutzomyia migonei*), make up the epidemiological context of the disease in VL endemic areas in Brazil (Brasil, 2021). Although a consensus has yet to be reached about the role of felines in the epidemiological cycle of VL (Silveira et al., 2015; Pennisi & Persichetti, 2018; Nascimento et al., 2022), given xenodiagnosis studies that have shown that cats can be infectious for VL competent vectors (Maroli et al., 2007; Silva et al., 2010), these animals have been suggested as a secondary reservoir for the parasite in VL endemic areas (Pennisi et al., 2015; Silveira et al., 2015; Pennisi & Persichetti, 2018; Asfaram et al., 2019; Nascimento et al., 2022). Nevertheless, cats play an important role in the one-health approach to leishmaniosis due to the species’ social status as companion animals (Pennisi, 2015). Thus, the importance of *Leishmania* spp. infection in cats has been gaining increasing attention, especially considering the large number of cats taking up space previously occupied by dogs in households over the last few decades (Otranto, 2015; Pennisi, 2015).

The first report on infection by *Leishmania* spp. in domestic cats (*Felis catus*) was published more than a century ago (Sergent et al., 1912), and cats were initially believed to be resistant to *Leishmania* spp. infection (Pennisi et al., 2015). However, in recent years, case reports and epidemiological studies about cats infected by different *Leishmania* spp. have been increasingly documented around the world, particularly in Brazil (Pennisi et al., 2015; Oliveira et al., 2015; Benassi et al., 2017; Alves-Martin et al., 2017; Pennisi & Persichetti, 2018; Asfaram et al., 2019; Leonel et al., 2020; Nascimento et al., 2022). The occurrence of feline leishmaniosis in Brazil is estimated to have increased by up to 8%, considering both serological and molecular surveys (Asfaram et al., 2019), and to date, three *Leishmania* spp. have been reported to infect cats in the country: *Leishmania braziliensis*, *Leishmania amazonensis* and *Leishmania infantum* (Savani et al., 2004; Schubach et al., 2004; Souza et al., 2005; Nascimento et al., 2022) the latter being the most commonly described (Nascimento et al., 2022). Feline leishmaniosis (FeL) caused by *L. infantum* infection in cats was described in Brazil for the first time in 2004 (Savani et al., 2004), and since then *L. infantum* infection has been described in cats with (Costa et al., 2010) and without clinical signs (Oliveira et al., 2015; Benassi et al., 2017).

Although numerous cases of FeL have been reported in the last 20 years, many aspects of the disease in cats are still unclear (Pennisi et al., 2015; Pennisi & Persichetti, 2018; Asfaram et al., 2019). One of them, knowledge about clinical and pathological changes caused by FeL in cats, is limited and based only on case reports (Pennisi et al., 2015). So far, there are only pointwise descriptions of clinical signs and physiological changes, but no standards or categories of infection have been established that are applicable to cats, resulting in the use of information obtained from canine studies (Pennisi et al., 2015; Pennisi & Persichetti, 2018). This is attributed mostly to the fact that, despite the increase in reported cases, the number of infected and sick cats is low and there are areas endemic for canine leishmaniosis (CanL) and human VL, with very few or no records of FeL cases (Nascimento et al., 2022). However, despite the various similarities between FeL and CanL, there are relevant differences between dogs and cats that cannot be ignored (Pennisi & Persichetti, 2018). In view of the above, this study evaluates and compares clinical, hematological, and biochemical parameters of cats naturally infected and uninfected by *L. infantum* in an area endemic for VL in Brazil, seeking to bring together a larger body of data regarding the clinical pathology of FeL.

## Material and Methods

### Study area and sample collection

Blood, serum, and conjunctival swabs (CS) were collected from 166 cats, in partnership with two animal shelters in the city of Ilha Solteira, state of São Paulo, Brazil (51° 06’ 35” W and 20° 38’ 44” S). The sample was limited to the number of cats living in the shelters at the time of sampling.

### Veterinary clinical examination

During sampling, the cats were examined by a veterinarian, who checked several parameters, such as mucosa color, nutrition status, abdominal and lymph node alterations (lymphadenopathy), and ocular and dermatological changes (alopecia, dermatitis, onychogryphosis and conjunctivitis). All the signs observed by the vet in each cat were recorded on an individual clinical chart. In addition, blood and serum from each animal were subjected to biochemical and hematological analysis.

#### Biochemical and hematological analysis

The hematology of the blood samples from all the cats was performed in a Mindray BC-2800 Vet^®^ hematology analyzer. A differential leukocyte count and cell morphology evaluation were performed on blood smears stained with Rapid Panoptic^®^. The hepatic and renal function of each animal were assessed based on measurements of alanine aminotransferase (ALT/GPT Liquiform Vet^®^, LabTest, ref. 1008-4/30), aspartate aminotransferase (AST/GOT Liquiform^®^, LabTest, ref. 109-4/30), urea (UREA UV Liquiform^®^, LabTest, ref. 104-4/50), and creatinine (CREATININA K^®^, LabTest, ref. 96-300), using a Mindray BS-120^®^ biochemistry analyzer calibrated with Calibra H^®^ (LabTest, ref.80) and quality controlled with Qualitrol 1H^®^ (LabTest, ref.71). Total plasma protein, albumin and alkaline phosphatase concentrations were also evaluated, and all the results were compared with the reference values for cats (Feldman et al., 2000; Kaneko et al., 2008).

### Serological testing

The serological evaluation of anti-*Leishmania* spp. antibodies was performed by indirect enzyme-linked immunosorbent assay (ELISA), according to Costa et al. (2010). Determination of the optical density (OD) for the cut-off point and classification of the ELISA levels (EL) (OD = ≥ 0.220, EL ≥ 3) were carried out as described by Oliveira et al. (2008). An indirect immunofluorescent antibody test (IFAT) was performed according to a previous study by Vides et al. (2011), using 1:40 dilution as the cut-off point.

### Molecular diagnosis

#### DNA extraction

DNA was extracted from blood samples using a DNeasy^®^ Blood & Tissue kit (QIAGEN), following manufacturer’s instructions. DNA from conjunctival swabs (CS) was extracted using the salting-out technique described by John et al. (1991), modified by Lahiri & Nurnberger (1991). The extracted DNA was stored at -20º C for subsequent conventional (cPCR) and quantitative (qPCR) PCR analysis.

#### Endogenous control

To exclude false negatives stemming from PCR errors or sample degradation, all the blood and CS DNA samples were subjected to a qPCR for the mammalian ß-actin endogenous gene, as described by Manna et al. (2006). A sample of feline DNA was used as positive control, while ultrapure sterile water was used as negative control. The analyses were carried out following the guidelines of the Minimum Information for Publication of Quantitative Real-Time PCR Experiments (MIQE) (Bustin et al., 2009).

#### cPCR for *Leishmania* spp. kDNA and Trypanosomatidae rDNA

DNA extracted from blood and CS samples was subjected to cPCR amplification of the conserved region of *Leishmania* spp. kinetoplast minicircle DNA (kDNA), as described by Rodgers et al. (1990). The kDNA cPCR positive samples were also tested using primers targeting the internal transcribed spacer region 1 (ITS-1) of the ribosomal DNA (rDNA) for Trypanosomatidae (Feldman et al., 2000). All the cPCR reactions were performed in a Veriti^®^ thermal cycler (Applied Biosystems). A DNA sample extracted from *L*. *infantum* (MCAN/BR/1984/CCC-17.481) was used as positive control, and ultrapure sterile water was used as negative control. The ITS-1 cPCR positive amplified products were subjected to DNA sequencing to identify the *Leishmania* spp.

#### qPCR to *L*. (*L*.) *infantum* kDNA

To detect *L. infantum* kDNA in cats, a qPCR was performed as proposed by Francino et al. (2006), using a LightCycler^®^ 480 II thermal cycler (Roche Diagnostics). A DNA sample extracted from *L*. *infantum* (MCAN/BR/1984/CCC-17.481) was used as positive control, while ultrapure sterile water was used as negative control. The analyses were performed as described by Bustin et al. (2009).

### Parasitological diagnosis

A parasitological diagnosis (PA) was performed using slides containing smears taken from lymph nodes and bone marrow aspirates. In some cases, imprints of lesions and/or punctures of dermal nodules were analyzed. The aspirates and imprint smears were stained using a Rapid Panoptic^®^ kit, as per the manufacturer’s instructions. The slides were examined under an optical microscope (400-1000 magnification). The stained blood and imprint smears that presented an amastigote form of *Leishmania* spp. were considered positive. A smear was considered negative if the parasite was not identified in at least 100 visual fields.

### Data analysis

#### DNA sequencing

The ITS-1 PCR-positive amplified products were purified using a GE Healthcare kit (Illustra®, GFX PCR DNA and GEL Band Purification Kit, catalog number 28-9034-70), following the manufacturer’s instructions. The sequences were analyzed by the DNA Sequencing Service of the Human Genome and Stem Cell Research Center at the Institute of Biosciences (IB), USP. Chromatograms obtained with forward and reverse primers were assessed using Sequence Scanner software 2.0 v2.2 (Applied Biosystems). The sequences were aligned using the ClustalW system available in the BioEdit version 7.1.11 Sequence Alignment Editor to generate consensus sequences. Contig sequences were compared to accession numbers in deposited GenBank to find similarities and mismatches between ITS1 sequences of *Leishmania* spp. isolates and NCBI-GenBank *Leishmania* spp. strains, using the Basic Local Alignment Search Tool (BLAST) (Ye et al., 2006).

#### Nonparametric statistical analysis

A non-parametric statistical analysis was used to evaluate the performance of the diagnostic methods in cats. The agreement index between the diagnostic methods was assessed using the Kappa index (κ) and interpreted according to Landis & Koch (1977), where κ <0.4 is considered to be poor agreement; 0.41 ≤ κ ≤ 0.6 is accepted as moderate agreement; 0.61 ≤ κ ≤ 0.80 is considered good agreement; and κ> 0.8 is accepted as excellent agreement.

The chi-square test with a significance level of 5% was calculated using R version 3.3.0 software (R Core Team, 2016) in order to assess the associations between the positivity of cats classified as with and without clinical signs in each PCR protocol (13A/13B, LITSR/L5.8S), as well as in the serological (ELISA and IFAT) and PA.

#### Statistical analysis of hematological and biochemical parameters

**Figure 1:**
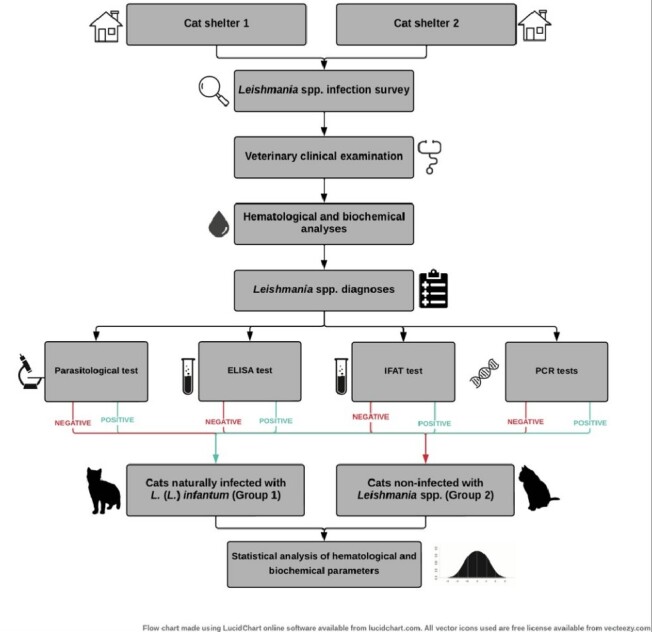
Experimental study design showing the formation of the groups: G1 (six cats *Leishmania* (*Leishmania*) *infantum* positive by PCR and sequencing), and G2 (six cats *Leishmania* spp. negative by all the tests).

Two groups were established for the statistical analysis of the hematological and biochemical parameters of *L. infantum* infected and *Leishmania* spp. non-infected cats, according to the serological, molecular and/or parasitological diagnostic results obtained in the survey of all the cats from the shelters (Figure 1). The hematological and biochemical parameters were evaluated by comparing the mean values of each parameter from each experimental groups (G1 and G2) using Student’s *t* test, with 95% confidence (p< 0.05). The student's t-test was used to compare the means of two independent variables.

The cats of both groups (G1 and G2) were all kept at the same shelter, living in the same conditions as before the beginning of the study, i.e., no changes were made in housing or food. In addition, to exclude the possibility of co-infection with FIV (feline immunodeficiency virus) and FeLV (feline leukemia virus), all the cats in G1 and G2 were tested using the SNAP FIV/FeLV Combo Test diagnostic kit (IDEXX Laboratories, Markham, Ontario), following the manufacturer’s instructions, which indicated they were negative for FIV/FeLV.

## Results

One hundred and sixty-six cats were subjected to clinical, serological, molecular, and PA (Figure 1). During the veterinary clinical examination, 54.8% (91/166) of the cats exhibited clinical abnormalities, and 90.1% of them (82/91) tested positive for anti-*Leishmania* spp. antibodies by at least one serological method (Table 1).

**Table 1:** Serological, molecular, and parasitological tests for the detection of *Leishmania* spp. in blood from cats with and without clinical signs of infection.

**Clinical examination**	**Positive Serological Tests**		**Positive cPCRs**		**Parasitological Positive**
	**ELISA**	**IFAT**	**13A/13B**	**LITSR/L5-8S**	
**Without clinical signs (N= 75)**	4b	28a	0c	0^c^	0^c^
**With clinical signs (N= 91)**	21^b^	61^a^	6^c^	6^c^	3^c^
**Total (N=166)**	25^b^	89^a^	6^c^	6^c^	3^c^

Proportion test (chi-square). 13A/13B: kDNA *Leishmania* spp.; LITSR/L5-8S: SSU-rDNA for Trypanosomatidae.

a, b, c different lower-case letters indicate statistical significance between columns at the 5% probability level (p ≤ 0.05).

In the clinical examination, 41.0% (68/166) of the cats presented skin lesions (such as papules, nodules, ulcers, erythema, and alopecia) on the body and especially on the head (face, nose, or ears). Another 29.5% (49/166) showed weight loss (body score 1), while 10.2% (17/166) of cats sampled exhibited enlarged lymph nodes. Albeit to a lesser extent, ocular lesions were also observed in 5.4% (9/166), cachexia in 4.2% (7/166) and diarrhea in 3.0% (5/166) of the cats.

As for the serological methods, 15% (25/166) and 53.6% (89/166) of the cats tested positive by ELISA and IFAT, respectively, and 12.0% (20/166) tested positive by both techniques. Regarding ELISA, the OD of positive cats ranged from 0.222 to 0.973 (EL = 3 to 7). By IFAT, the antibody titers of positive cats were 1:40 (27/89), 1:80 (32/89), 1:160 (19/89), 1:320 (7/89), 1:640 (1/89) and 1:1280 (2/89).

**Figure 2:**
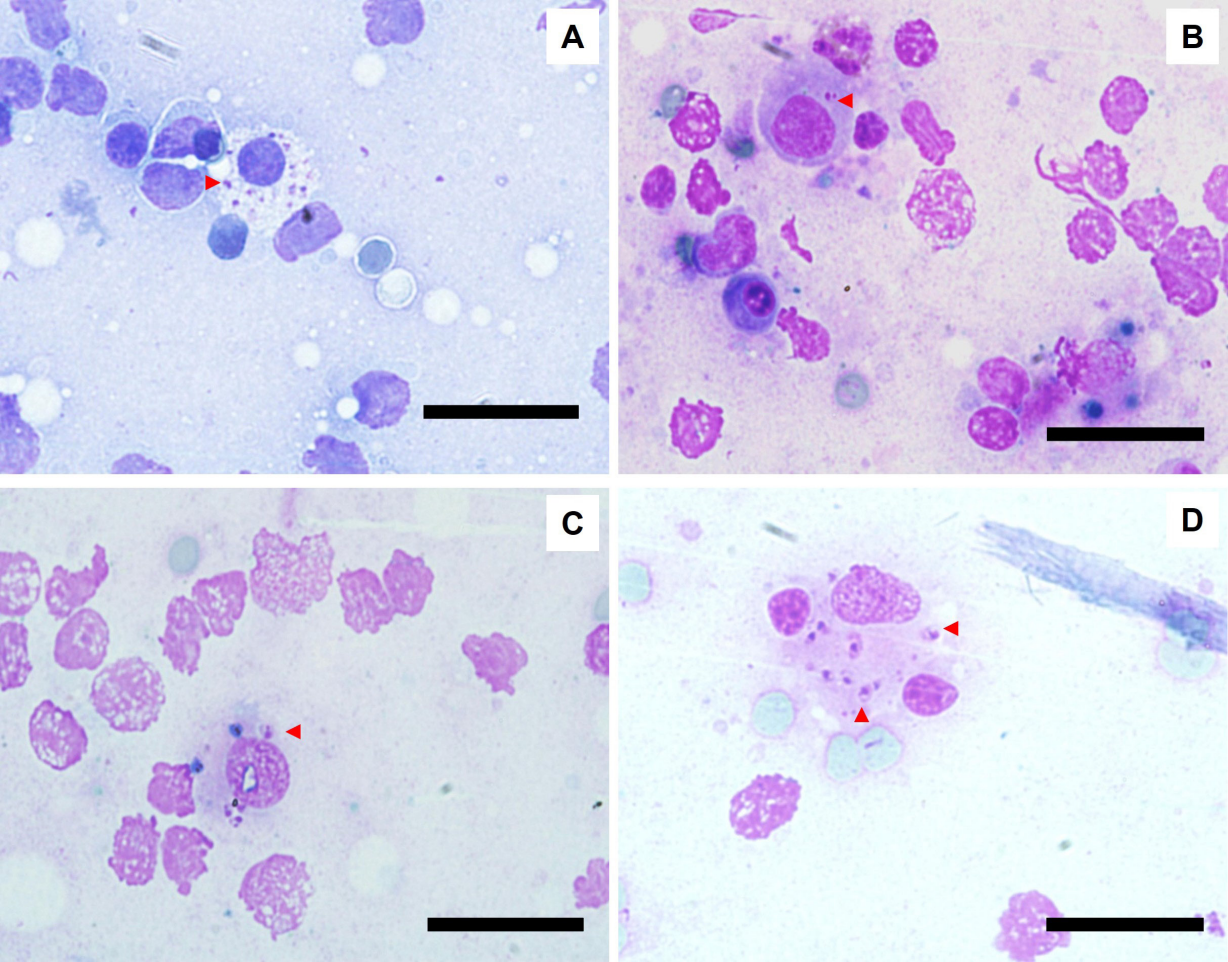
*Leishmania* spp. amastigotes inside macrophages (red arrow) visualized in a popliteal lymph nodes smear stained with a Rapid Panoptic^®^ kit. Caption: cat 2 (A) and cat 3 (B, C and D). Bar = 50μm, 100x objective lens.

As for the molecular diagnosis, the DNA from all the blood and CS samples was positive for mammalian ß-actin gene (endogenous control). This confirmed the quality of the DNA extraction process and proved that there were no inhibitors and/or false negative results in the molecular analysis. With respect to *Leishmania* spp. DNA, 3.6% (6/166) of the blood and CS samples were positive by PCR and qPCR. Analysis by direct sequencing and ITS-1 amplicons of all samples revealed a 100% match with *L. infantum* (similarity with sequences in GenBank^®^ under accession number KY379078.1). All these PCR positive cats were seropositive, and 3 cats presented *Leishmania* spp. amastigote forms in lymph node smears (Figure 2).

Among all the tests employed in this study, a significantly higher number of positive cats displaying clinical signs (61/91) were detected by IFAT than by the other tests (p≤0.05), and only cats with clinical signs tested positive by parasitological (PA) methods and/or PCR (Table 1).

Only the concurrence between PA vs. cPCR or qPCR (κ = 0.6584) was considered good by the Kappa index. ELISA vs. IFAT (κ = 0.1513), ELISA vs.PA (κ = 0.0313), IFAT vs. PA (κ = 0.2309) and IFAT vs. (cPCR and qPCR) (κ = 0.0628) presented poor concurrence and ELISA vs. cPCR or qPCR (κ = 0.4298) moderate concurrence.

**Figure 3:**
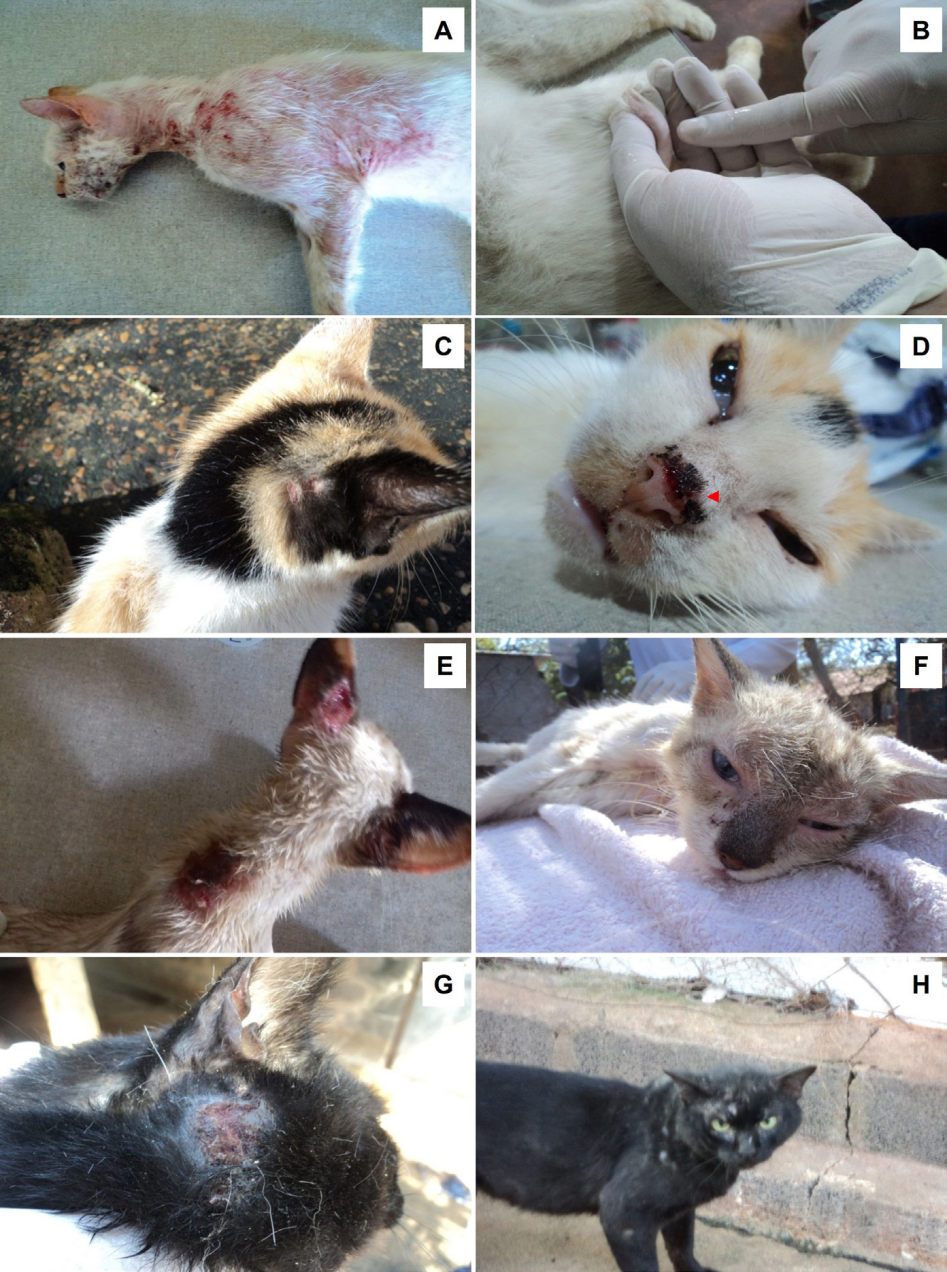
Clinical signs in cats naturally infected with *Leishmania* (*Leishmania*) *infantum*. Caption: alopecic and erythematous area with crusty ulcerations on the face and body of cat 02 (A); enlarged lymph node in which amastigotes of the parasite were identified in cat 02 (B); alopecic, erythematous and circular area on ear and nose of cat 03 (C and D); ulcerative lesions on ear and neck of cat 04 (E); conjunctivitis in cat 04 (F); crusty ulcerative lesion on the head and alopecic areas on the face and body of cat 05 (G and H).

Based on these results, two groups were stablished for the statistical analysis of hematological and biochemical parameters of *L*. *infantum* infected (G1) and *Leishmania* spp. non-infected cats (G2). The number of cats in group G1 was defined by the total number of *L. infantum* cats positive by amplicon sequencing, and G2 was established as a control group with an equal number of uninfected cats. Cats of both groups were also FIV and FeLV negatives, as described in Table 2. All the cats in G1 group exhibited clinical signs suggestive of FeL (Table 2, Figure 3). Table 3 lists the results of hematological and biochemical parameters. As can be seen, the cats in G1 showed significantly lower platelet counts (p = 0.0062) and higher erythrocyte counts (p = 0.0063) than those in G2. Leukocytes were higher in G2 (p = 0.0140), specifically neutrophils (p = 0.0410) and lymphocytes (p = 0.0495), but only platelet counts were outside the reference range for the species (Table 3). As for their biochemical parameters, *L*. *infantum* positive cats showed augmented total plasma protein (p = 4.4832e-06) and low albumin levels (p = 0.0065) (Table 3). In addition, group GI showed lower aspartate aminotransferase enzyme levels (p = 0.0025) than G2 (Table 3). All these parameters fell outside the reference range for cats (Table 3). Also, hyperproteinemia and hypoalbuminemia were more evident in cats with severe clinical signs that tested positive by parasitological methods.

**Table 2:** Groups of cats infected (G1) and non-infected (G2) with *L.* (*L.*) *infantum* according to serological, molecular, and parasitological diagnosis selected for clinical, hematological and biochemical evaluation.

**Group**	**Cats**	**Clinical signs**	**FIV/** **FeLV**	**Serological diagnosis**		**Molecular diagnosis**			**PA**	**Sequencing**
				**ELISA**	**IFAT**	***Leishmania* spp. kDNA** ¶	***L.* (*L.*) *infantum* kDNA** †	**Trypanosomatidae** **rDNA** ^¶^		
**G1**	1	Weight loss (score 1) and skin lesions on nose	N	+ / 5	+ / 1:40	+	+	+	+	*L.* (*L.*) *infantum*
	2	Weight loss (score 1), lymph node enlargement, skin lesion on ear and body associated with alopecia	N	+ / 6	+ / 1:80	+	+	+	+	*L.* (*L.*) *infantum*
	3	Weight loss (score 1), skin lesions on nose and body	N	+ / 3	+ / 1:80	+	+	+	+	*L.* (*L.*) *infantum*
	4	Weight loss (score 1), lymph node enlargement, skin and ocular (conjunctivitis) lesions	N	+ / 4	+ / 1:320	+	+	+	N	*L.* (*L.*) *infantum*
	5	Weight loss (score 1) and skin lesions	N	+ / 6	+ / 1:80	+	+	+	N	*L.* (*L.*) *infantum*
	6	Weight loss (score 1) and alopecia lesions	N	+ / 3	+ / 1:80	+	+	+	N	*L.* (*L.*) *infantum*
**G2**	1	No clinical signs	N	N / 2	N	N	N	N	N	NA
	2	No clinical signs	N	N / 2	N	N	N	N	N	NA
	3	No clinical signs	N	N / 2	N	N	N	N	N	NA
	4	No clinical signs	N	N / 1	N	N	N	N	N	NA
	5	No clinical signs	N	N / 0	N	N	N	N	N	NA
	6	No clinical signs	N	N / 0	N	N	N	N	N	NA

+ positive; N: negative; NA: not applicable; ELISA: enzyme-linked immunosorbent assay with respective ELISA levels; IFAT: indirect immunofluorescence test with respective antibody titers; PA: parasitological diagnosis

¶ based on conventional PCR;

† based on quantitative PCR.

**Table 3:** Hematological and biochemical parameters of cats infected (G1) and non-infected (G2) with *L*. (*L*.) *infantum*.

**Parameters**	**Reference range** ¶	**Infected group**	**Non-infected group**	***p*-value**
Red blood cells (10^6^/μL)	5 - 10	8.07 ± 0.51a	7.03 ± 0.46b	***p* < 0.01**
HCT (%)	24 - 45	33.76 ± 3.21^a^	32.26 ± 2.30^a^	0.3927
Hemoglobin (g/dl)	8 - 15	10.14 ± 0.69^a^	10.36 ± 0.24^a^	0.4708
MCV (fl)	39 - 55	44.32 ± 4.29^a^	46.00 ± 4.49^a^	0.5444
MCHC (%)	30 -36	30.1 ± 0.87^a^	32.26 ± 2.64^a^	0.1154
Platelet count (10^3^/μL)	230 - 680	122.4 ± 16.07^b^	265.5 ± 88.25^a^	***p* < 0.01**
White blood cells (10^3^/μL)	5.5 -19	13.78 ± 4.09^b^	21.25 ± 4.03^a^	***p* < 0.05**
Eosinophils (10^3^/μL)	0 -1.5	0.98 ± 0.50^a^	1.73 ± 0.22^a^	0.06332
Neutrophils (10^3^/μL)	2.5 - 12.5	10.17 ± 3.37^b^	14.15 ± 2.21^a^	***p* < 0.05**
Lymphocytes (10^3^/μL)	1.5 - 7	2.58 ± 1.04^b^	5.11 ± 2.54^a^	***p* < 0.05**
Monocytes (10^3^/μL)	0 - 0.85	0.40 ± 0.36^a^	0.25 ± 0.24^a^	0.4293
Urea (mg/dl)	42.8 - 64.2	97.6 ± 16.51^a^	97 ± 14.01^a^	0.9494
Creatinine (mg/dl)	0.8 - 1.8	1.04 ± 0.21^a^	1.46 ± 0.36^a^	0.05226
ALP (U/L)	7 - 80	15.78 ± 1.56^a^	13.97 ± 2.50^a^	0.1957
ALT (U/L)	06 - 83	36 ± 12.94^a^	39.5 ± 20.63^a^	0.7506
AST (U/L)	26 - 43	21.6 ± 4.97^b^	38 ± 7.56^a^	***p* < 0.01**
Total Plasm Protein (g/L)	54 - 78	92.6 ± 3.28^a^	72.5 ± 3.50^b^	***p* < 0.01**
Albumin (g/dl)	2.1 - 3.3	1.54 ± 0.11^b^	2.26 ± 0.44^a^	***p* < 0.01**

HCT: hematocrit; MCV: mean cell volume; MCHC: mean corpuscular hemoglobin concentration; ALP: alkaline phosphatase; ALT: alanine aminotransferase; AST: aspartate aminotransferase.

¶ Reference values as proposed by Feldman et al. (2000) and Kaneko et al. (2008);

^a/b^: different lower case letters indicate statistical difference between columns at 1% (p ≤ 0.01) or 5% probability (p ≤ 0.05).

## Discussion

To fulfill the goal of this study, 166 cats were analyzed by serological, molecular and parasitological methods. Among them, 15% presented anti-*Leishmania* spp. antibodies by ELISA and 53.6% by IFAT. Some cats testing positive by ELISA reached up to 4-fold higher antibody levels than the cut-off limit, as previously observed in other studies (Solano-Gallego et al., 2007; Vides et al., 2011). Cats positive by IFAT showed antibody titers up to 1:1280; higher than reported in previous studies involving cats naturally infected with *Leishmania* spp. (Vides et al., 2011; Alves-Martin et al., 2017; Leonel et al., 2020).

The number of seropositive cats identified in this study was statistically similar to that previously described in the same area (Alves-Martin et al., 2017). Seropositive cats are common in areas endemic for canine leishmaniosis (CanL), where surveys have revealed anti-*Leishmania* spp. seroprevalence rates ranging from 0% to more than 60% (Pennisi et al., 2015). Cohabitation of the cats in this study with infected dogs and sand flies may have favored exposure of the cats to the parasite. Other authors have also reported high serological positivity in sheltered cats (Baneth et al., 2020; Leonel et al., 2020) and a comparison between sheltered and owned cats observed a higher seroprevalence in the former (Matos et al., 2018). However, serological results should be interpreted with caution, as these tests are unable to distinguish past from current infections and the possibility of cross-reactions cannot be ruled out. The poor concurrence between serological techniques (ELISA vs. IFAT) corroborates the findings of an earlier study in a population of cats in a CanL endemic area (Vicente et al., 2012). As also described in dogs, discrepancies are known between serological tests as IFAT and ELISA (Pennisi & Persichetti, 2018). A cutoff of 1:80 has been recommended for serodiagnosis by IFAT for dogs and cats in Europe (Pennisi et al., 2015; Persichetti et al., 2017) and this could improve the agreement between ELISA and IFAT. But here in this study, a sick cat diagnosed positive by PCR and PA had an antibody titer of 1:40 on IFAT, so the cut-off point was kept at 1:40. Although many cats tested positive by IFAT, 28 of these cats showed no clinical signs of FeL. Persichetti et al. (2017) using a Bayesian analysis without a gold standard concluded that IFAT (cut-off 1:80) was more sensitive than ELISA to detect subclinical or early infections, while ELISA was better for diagnosing clinical leishmaniosis when compared with IFAT.

Overall, different techniques used for the diagnosis of *Leishmania* spp. infection in cats do not always provide convergent results, and concurrence can vary from poor to moderate (Alves-Martin et al., 2017; Pennisi & Persichetti, 2018). According to Pennisi et al. (2015), the same technique may produce different values ​ due to differences in levels of endemism, specific characteristics of the animals under study, and differences in the diagnostic methodology. Direct diagnosis enables the detection of *Leishmania* spp. with high specificity and variable sensitivity, since factors such as the degree of parasitism and types and processing of biological samples and coloring can interfere with the sensitivity (Chatzis et al., 2014; Alves-Martin et al., 2017). In this study, the detection of *Leishmania* spp. amastigote forms by PA and parasite DNA by molecular tests was low; however, the agreement between PA and cPCR for *Leishmania* spp. plus PA and qPCR for *L*. *infantum* was considered good. Thus, the combined use of the two techniques (molecular and serological) is essential to ensure the accurate diagnosis of infection in cats, using PCR to amplify the parasite DNA for the sequencing and determination of the infecting species (Silveira et al., 2015). Therefore, the diagnosis of FeL in this study was reached using different methods, and PCR sequencing matching 100% with *L. infantum* was the reference point to select infected cats for the G1 group. In addition, the cats in G1 and G2 were all FIV and FeLV negatives.

Molecular investigations of *Leishmania* spp. DNA in cats do not usually differ, in methodological terms, from dogs (Pennisi, 2015), but in areas endemic for CanL, molecular positivity rate in cats is lower than in dogs (Otranto, 2015; Pennisi, 2015). In our study, we also observed this difference, since the frequency of CanL in the city of this study was estimated to be 13.1% by molecular tests (Pereira et al., 2016), in comparison to the 3.6% PCR positivity among cats in the present study.

According to the literature, skin lesions and lymph node enlargement are the most frequent clinical signs of leishmaniosis in cats, regardless of the species of *Leishmania* infection (Silveira et al., 2015; Pennisi et al., 2015). In addition, the head, ears and nose are the most affected areas, presumably due to the tendency for phlebotomine sand flies to bite hairless areas (Simões-Mattos et al., 2004; Silveira et al., 2015). Here, skin lesions were seen in 41.0% (68/166) of the cats and other signs consistent with FeL, such as weight loss (body score 1) and lymph node enlargement were also observed. These signs are non-specific and common to other diseases affecting cats, such as immunosuppression from retroviruses and fungal infections (Pennisi et al., 2015) and despite the majority being seropositive, only 6 were positive by direct diagnosis. Suggesting that most cats have been exposed to *Leishmania*, but a minority are infected.

All the above-described clinical signs were observed in G1 (Table 2, Figure 3). In addition, one infected cat shoed an ocular lesion (Table 2).

The hemogram showed a significantly lower platelet count in G1 than in G2 (p<0.001) (Table 3). Thrombocytopenia is a sign of CanL and may be associated with the clinical stage of the disease and the presence of IgM and IgG anti-platelet antibodies (Ciaramella et al., 2005; Terrazzano et al., 2006; Cortese et al., 2009; Braz et al., 2015). Thus, thrombocytopenia in CanL may result from changes in vessel walls due to vasculitis caused by the deposition of immune complexes, which may be linked to the presence of antiplatelet immunoglobulins or to changes in thrombocytopoiesis; moreover, it is related to renal or hepatic failure in clinically affected (Ciaramella et al., 2005; Terrazzano et al., 2006). In cats, the diagnosis of antibody-mediated thrombocytopenia is still unknown, mainly due to the lack of a sensitive and specific assay for the detection of antiplatelet antibodies. This makes it impossible to speculate about the existence of significant differences in anti-platelet antibody binding activity between infected (G1) and non-infected (G2) cats.

In group G1, hyperproteinemia with hypoalbuminemia was a significant biochemical parameter that differed from the *Leishmania* spp. negative group (G2) (p<0.001) (Table 3). These biochemical changes, which are commonly observed in CanL due to a high level of antibodies, mainly anti-*Leishmania* spp. IgGs, provide a disease marker for dogs in VL endemic areas (Medeiros et al., 2008; Freitas et al., 2012; Braz et al., 2015; Montargil et al., 2018). Thus, with respect to dogs, progression of the disease is accompanied by a strong humoral response, as well as downregulation of the cellular response (Pinelli et al., 1994).

Consistent data regarding the immune response in FeL are scarce, but in a study of 100 sheltered cats in the same endemic area in Brazil, 60 were positive by the Montenegro Skin Test (MST). However, only 5 cats in the same group were sick with positive PCR and PA for *L. infantum* and of these, only 1 was MST positive (Alves et al., 2022). In humans with VL, MST is negative during the acute phase of the disease but becomes positive after resolution of clinical symptoms (Pearson & Sousa, 1996). In CanL, 81% of the Ibizan Hound dogs (CanL resistant) were MST positive, while only 48% of dogs of other breeds in the same endemic area were positive. Therefore, Ibizan Hounds are considered to have a more uniform cellular response and are more resistant to *L. infantum* infection than dogs of other breeds (Solano-Gallego et al., 2000). Maybe in cats, dysregulation of the cellular response may also be related to the progression of the disease. Here, *L. infantum* positive cats in G1 showed significantly higher serum levels of IgG antibodies by ELISA and IFAT than did healthy cats, while PCR was effective in the detection of *Leishmania* spp. only in samples from cats with clinical signs. All the cats testing positive by PA and PCR exhibited clinical signs consistent with FeL and were also seropositive. Most of the cats in the current study can be considered as exposed to *Leishmania*, but in a small number it was possible to detect the parasite.

## Conclusions

The hematological and biochemical data described here indicate a significant association between FeL caused by *L. infantum*, low platelet counts, and hyperproteinemia with hypoalbuminemia. This suggests that in VL endemic areas, cats showing clinical signs consistent with FeL (such as skin lesions, weight loss, enlarged lymph nodes or eye lesions) associated with hematological (low platelet count) and biochemical changes (hyperproteinemia and hypoalbuminemia) should be subjected to a differential diagnosis of FeL caused by *L. infantum* infection. In addition, antibodies against the parasite were detected in both healthy and clinically affected cats; however, the presence of clinical signs enhances detection by serological, parasitological, and molecular tests.
